# Alpha-Lipoic Acid in Early-Stage Alcohol-Related Brain Damage in Rats: A Comparative Pilot Study

**DOI:** 10.3390/molecules30194007

**Published:** 2025-10-07

**Authors:** Hristian Staykov, Stela Dragomanova, Yordan Hodzhev, Valya Grigorova, Borislav Minchev, Diamara Uzunova, Ani Georgieva, Inna Sulikovska, Katerina Todorova, Elina Tsvetanova, Almira Georgieva, Miroslava Stefanova, Pendar Valadbeigi, Reni Kalfin, Rumen Nikolov, Lyubka Tancheva

**Affiliations:** 1 Department of Pharmacology and Toxicology, Faculty of Medicine, Medical University of Sofia, Zdrave 2 St., 1431 Sofia, Bulgaria; drhristianstaykov@gmail.com (H.S.); pendar.waladbaigi@gmail.com (P.V.); rnikolov@medfac.mu-sofia.bg (R.N.); 2 Department of Pharmacology, Toxicology and Pharmacotherapy, Faculty of Pharmacy, Medical University of Varna, 55 Marin Drinov St., 9000 Varna, Bulgaria; stela_dragomanova@abv.bg; 3 Department of Natural Sciences, New Bulgarian University, 21 Montevideo Blvd., 1618 Sofia, Bulgaria; jordanqvo@gmail.com; 4 National Center of Infectious and Parasitic Diseases, 26 Yanko Sakuzov Blvd., 1504 Sofia, Bulgaria; 5 Institute of Neurobiology, Bulgarian Academy of Sciences, 23 Acad. G. Bonchev St., 1113 Sofia, Bulgaria; vbgrigorova@abv.bg (V.G.); boby_al@mail.bg (B.M.); didi_uzunova1@abv.bg (D.U.); elina_nesta@abv.bg (E.T.); almirageorgieva@gmail.com (A.G.); mira_stefanova@mail.bg (M.S.); lyubkatancheva@gmail.com (L.T.); 6 Institute of Experimental Morphology, Pathology and Anthropology with Museum, Bulgarian Academy of Sciences, 25 Acad. G. Bonchev St., 1113 Sofia, Bulgaria; georgieva_any@abv.bg (A.G.); inna_sulikovska@ukr.net (I.S.); katerinagencheva@yahoo.com (K.T.); 7 Department of Healthcare, Faculty of Public Health, Healthcare and Sport, South-West University, 66 Ivan Mihailov St., 2700 Blagoevgrad, Bulgaria

**Keywords:** alpha-lipoic acid, ethanol, alcohol-related disorders, alcohol-related brain damage, neurodegeneration, neuroprotection, oxidative stress, dementia, memantine, rivastigmine

## Abstract

Alcohol misuse can lead to alcohol-related brain damage (ARBD), a condition linked to long-term cognitive impairment and considerable disease burden. The pharmacological characteristics of alpha-lipoic acid (ALA) make it a promising candidate for the treatment of ARBD. In this study, adult male Wistar rats were divided into eight experimental groups. Four groups received a 20% (*v*/*v*) ethanol–tap water solution ad libitum for 15 weeks to induce early-stage ARBD, while the remaining received only tap water. After 14 weeks, all groups were administered daily injections for one week with either ALA, rivastigmine, or memantine. Behavioral testing included the step-through passive avoidance and rotarod performance tests. Whole-brain biochemical analyses assessed acetylcholinesterase activity, brain-derived neurotrophic factor, and oxidative stress biomarkers. Brain weight, relative brain weight, and brain histopathological changes were also evaluated. Results showed that, similar to memantine and rivastigmine, ALA improved STL at both 24 h and 8 days and reduced ethanol-induced Purkinje cell damage. It also decreased lipid peroxidation levels by 44%, unlike the reference drugs, and superoxide dismutase activity by 33%, similar to them. No other significant changes were detected. Albeit several limitations, this is the first study comparing ALA with rivastigmine and memantine in this experimental context.

## 1. Introduction

Likely owing to its psychoactive effects, alcohol (ethanol) has been enjoyed worldwide both as a recreational beverage and to self-medicate, which has, however, not been without significant health risks and harms to its most (and even not-so) regular drinkers [1,2,3]. A systematic review and meta-analysis of 107 cohort studies (>4.8 million participants) reported increased all-cause mortality in women consuming ≥ 25 g/day and men ≥ 45 g/day of ethanol [4]. Globally, alcohol misuse ranked as the seventh-leading risk factor for premature death and disability (DALYs) in 2016 [5], and in 2019 caused about 2.6 million deaths worldwide, males being disproportionately affected at 2 million deaths compared to 600,000 deaths in females [6].

Alcohol misuse can damage nearly every organ in the body, including the brain [7]. Alcohol-related brain damage (ARBD) is an umbrella term that encompasses a spectrum of neurological and neuropsychiatric syndromes, directly or indirectly due to the neurotoxic effects of ethanol. ARBD is not dementia, and in some patients, it can be reversed after sustained abstinence from alcohol. If drinking is not stopped, however, ARBD can slowly progress to include a residual permanent cognitive impairment [8,9]. ARBD is thought to be underdiagnosed [9], but it has been estimated that 1 in 8 people who are dependent on alcohol may go on to develop it, with men being at higher risk due to more often misusing alcohol [10,11].

The mechanisms underlying ARBD are thought to involve a neuroinflammatory response mediated by neuroimmune factors, along with neurotoxic processes such as increased oxidative stress. Cholinergic dysfunction and altered neurotrophic activity also play a role. These responses may vary across brain regions. They are further influenced by the dose and duration of ethanol exposure, as well as by periods of withdrawal and abstinence [12,13]. There is currently no gold-standard treatment for ARBD, although various pharmacological agents have shown some promise [14].

**Figure 1:**
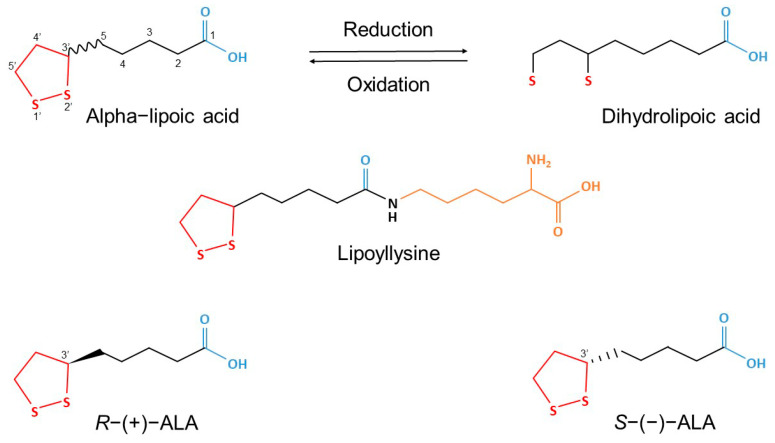
The chemical structure of racemic alpha-lipoic acid (ALA), its enantiomers (*R*−(+)−ALA and *S*−(−)−ALA), lipoyllysine (*R*−(+)−ALA, covalently bound to lysine residues, as present in plants and animals), and dihydrolipoic acid (DHLA) (Adapted from Wang et al., 2023 [21]) and Linus Pauling Institute at Oregon State University [22]).

One potential drug candidate is the molecule 5-(1,2-Dithiolan-3-yl)pentanoic acid (IUPAC), also known by its trivial names alpha-lipoic acid (ALA) and thioctic acid (Figure 1) [15]. It is a heterocyclic thia fatty acid that forms a redox couple with its biologically active reduced metabolite, dihydrolipoic acid (DHLA) [16,17]. Both ALA and DHLA are amphiphilic which facilitates their passage across the blood–brain barrier and allows distribution across various brain regions. In pharmaceutical and nutraceutical applications, ALA is typically marketed as a racemic mixture and is registered in different countries either as a medicinal product, a dietary supplement, or both. [18,19,20].

ALA has not yet been extensively investigated for its potential to mitigate ARBD. A limited number of studies have demonstrated that it can reduce voluntary ethanol self-administration and seeking behavior, and ethanol-induced increases in locomotor activity in mice [23,24], as well as protect against ethanol-induced neurotoxicity in the clonal mouse hippocampal cell line HT22 [25]. There is also data that it may increase the expression of hepatic fibroblast growth factor 21 (FGF21) [26,27] known to counteract ethanol-induced intoxication [28]. In addition to this, it has excellent antioxidant, anti-inflammatory, and neuroprotective effects that are largely opposed to the damaging effects of ethanol [29]. Their potential mechanisms of action are shown in Table 1.

ARBD can lead to structural alterations in vulnerable brain regions, which in turn cause impairments in memory and motor coordination, along with biochemical disturbances such as increased oxidative stress, reduced BDNF levels, and elevated AChE activity. Intervening early by targeting the toxicodynamic effects of ethanol can lead to improved therapeutic outcomes. Two current antidementia drugs [55] have shown efficacy in alleviating cognitive symptoms of ARBD in both preclinical and clinical studies: the N-methyl-D-aspartate (NMDA) receptor antagonist memantine [56,57,58,59,60,61,62] and the acetylcholinesterase (AChE) antagonist rivastigmine [63,64,65,66,67,68,69]. Building on this, the present comparative pilot study aimed to model early-stage ARBD in adult male Wistar rats via chronic administration of a 20% (*v*/*v*) ethanol–tap water solution for 15 weeks and to evaluate whether early intervention with alpha-lipoic acid could mitigate its damaging effects, compared to memantine and rivastigmine.

## 2. Results

### 2.1. Behavioral Phenotyping

#### 2.1.1. Step-Through Passive Avoidance Test

**Figure 2:**
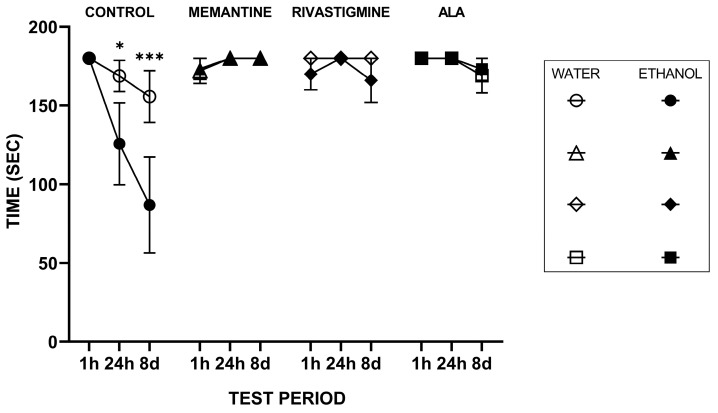
The effects of ALA, rivastigmine, and memantine on step-through latency (STL) in the WGs and EGs one hour (1 h), twenty-four hours (24 h), and eight days (8 d) after the first injection in a step-through passive avoidance test. Values represent mean ± SEM. The number of animals per group was *n* = 6 for all groups. Asterisks above the bars indicate significant differences (* *p* < 0.05 and *** *p* < 0.001). Statistical analysis was performed using a three-factor mixed linear model (MixedLM) with repeated measures, accounting for Control, Drug, and Time effects.

Figure 2 presents the mixed linear model (MixedLM) results for the step-through latency (STL) test. The Drug × Time interaction showed that the effect of drug treatment depended on the retention interval, driven largely by the saline condition, which in the ethanol-drinking animals displayed a marked decline in STL at 24 h (t = −2.51, df = 113, *p* = 0.014) and an even greater decline at 8 days (t = −3.85, df = 113, *p* < 0.001) compared to its 1 h performance. In addition, the Liquid × Drug × Time interaction (Water–Saline at 8 d, t(113) = 63.60, *p* = 0.03, Cohen’s d = −1.07) indicated that at 8 days, the E-S group had a significantly lower STL compared to the W-S group, suggesting that prolonged consumption of the ethanol–tap water solution (20%, *v*/*v*) hampers retention. This validates the experimental rat model of early-stage ARBD.

In contrast, the other drug treatments did not show such pronounced time-dependent declines (*p* > 0.05), implying that pharmacological intervention mitigated the retention loss observed in the saline-injected animals. These results indicate that the memory retention interval strongly modulated the effect of saline, and that ethanol consumption specifically impaired long-term memory performance compared to water, particularly in the absence of pharmacological intervention.

Notably, at 24 h, the improvement caused by ALA was equal to that of memantine and rivastigmine, and at 8 days it was similar to that of memantine and 4.2% greater than that of rivastigmine.

#### 2.1.2. Rotarod Performance Test

**Figure 3:**
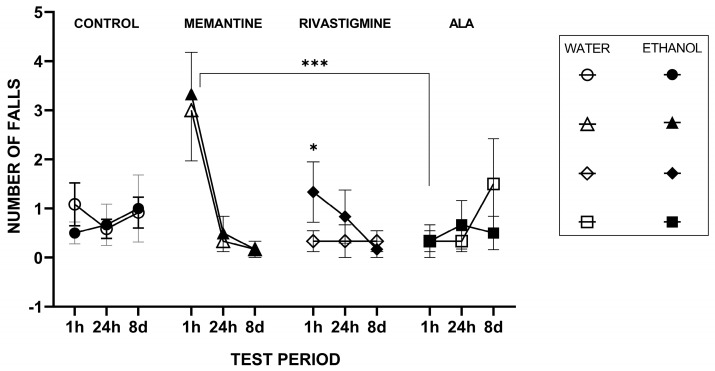
The effects of ALA, rivastigmine, and memantine on the number of falls in the WGs and EGs one hour (1 h), twenty-four hours (24 h), and eight days (8 d) after the first injection in a rotarod performance test. Values represent mean ± SEM. The number of animals per group was *n* = 6 for all groups. Asterisks above the bars indicate significant differences (* *p* < 0.05 and *** *p* < 0.001) based on post hoc comparisons following a three-way ANOVA (GraphPad Prism 9). The three-way ANOVA included Drug (saline, memantine, rivastigmine, ALA) × Ethanol (present/absent) × Time (training, 1 h, 24 h, 8 days) as fixed factors.

Figure 3 showed results from ANOVA with Time, Drug, and Ethanol as fixed factors and Animal as a blocking factor revealed a significant main effect of Drug (F(4117) = 6.11, *p* < 0.001, η^2^ = 0.39) but no main effect of Ethanol (*p* = 0.78) or Time (*p* = 0.134). Significant interactions were observed for Time × Drug (F(16,117) = 3.73, *p* < 0.001, η^2^ = 0.04) and Time × Ethanol (F(4117) = 3.32, *p* = 0.02, η^2^ = 0.15), while the Drug × Ethanol interaction was not significant (*p* = 0.51). The blocking factor Animal was also significant (*p* = 0.018), indicating substantial inter-individual variability in task performance. Post hoc Tukey HSD tests for the Time × Drug interaction indicated that at the 1 h time point, memantine produced significantly higher scores than ALA (mean difference = 2.94 g, *p* = 0.0001). No other pairwise drug–drug comparisons reached statistical significance after adjustment for multiple comparisons at any time point. Memantine treatment resulted in a markedly higher number of falls relative to ALA at the 1 h test; however, this difference was no longer evident at later time points.

### 2.2. Body Weight and Relative Brain Weight Measurement

#### 2.2.1. Body Weight Measurement

**Figure 4:**
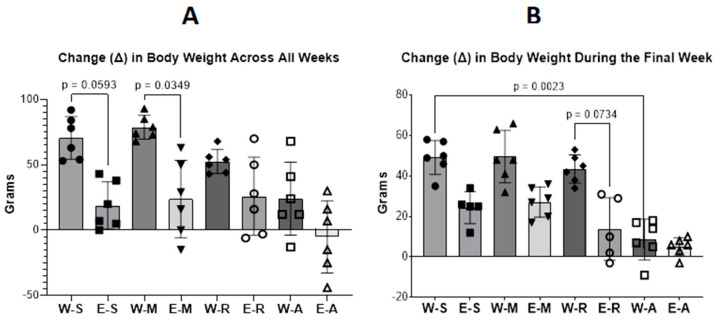
Changes (Δ) in body weight over the full 15-week experimental period (**A**) and during the final week (**B**), which corresponded to the injection period, in the WGs and EGs. Values represent mean ± SEM. One-way ANOVA with fixed factor Treatment was performed with GraphPad Prism. The number of animals per group was *n* = 6 for all groups. **Note**: The slight overall weight gain and the more pronounced change in the final week likely reflect stabilized growth in adult rats and temporary effects of hydration and feeding, rather than true health irregularities.

Statistical analysis of relative brain weight (Figure 4) showed a main effect of treatment (F(7, 46) = 12.11, *p* < 0.001, η^2^ = 0.65). A general number trend toward reduced body weight was observed across all weeks in all EGs. Further, multiple comparisons Dunnett’s test revealed that the decrease in the E-S group compared with the W-S group was approaching statistical significance (*p* = 0.06). A significant reduction was observed in the E-M group relative to the W-M group across all weeks but not during the final week per se. In contrast, during the final week, body weight in the E-R group showed a decline that approached statistical significance compared with the W-R group (*p* = 0.07), while the W-A group exhibited a significant decrease compared with the W-S group (*p* = 0.002).

#### 2.2.2. Relative Brain Weight Measurement

**Figure 5:**
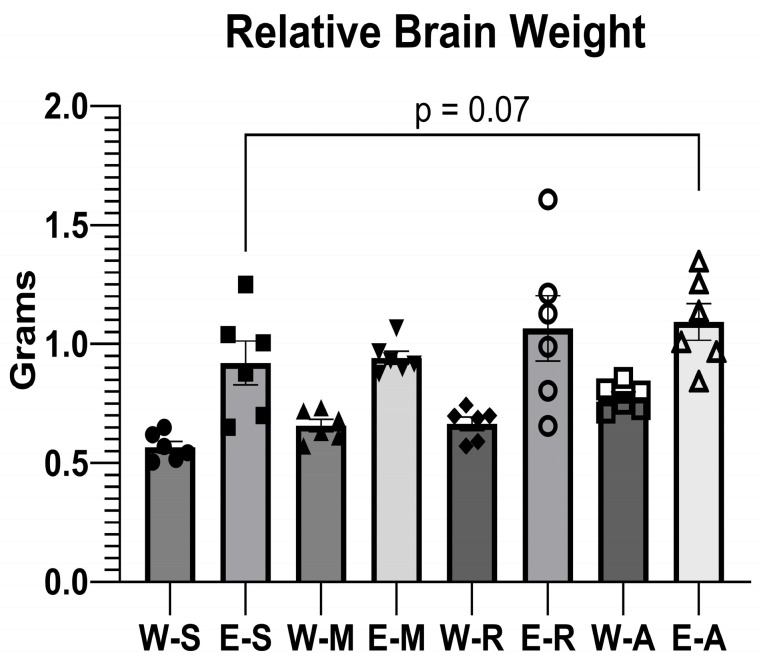
The effects of ALA, rivastigmine, and memantine on relative brain weight in the WGs and EGs. Values represent mean ± SEM. One-way ANOVA with fixed factor Treatment was performed with GraphPad Prism. The number of animals per group was *n* = 6 for all groups.

A general trend toward increased relative brain weight was observed in the EGs compared to their WG counterparts (Figure 5).

ALA administration in the E-A group caused an increase in relative brain weight that approached statistical significance (*p* = 0.07) when compared with the E-S group. The result was similar to those observed following treatment with memantine and rivastigmine.

It is important to note that the observed increase in relative brain weight reflects a denominator effect from reduced body weight in the ethanol-consuming groups, rather than a genuine increase in brain mass. Absolute brain weights did not differ significantly between groups when measured.

### 2.3. Histopathological Analysis of Brain Tissues

**Figure 6:**
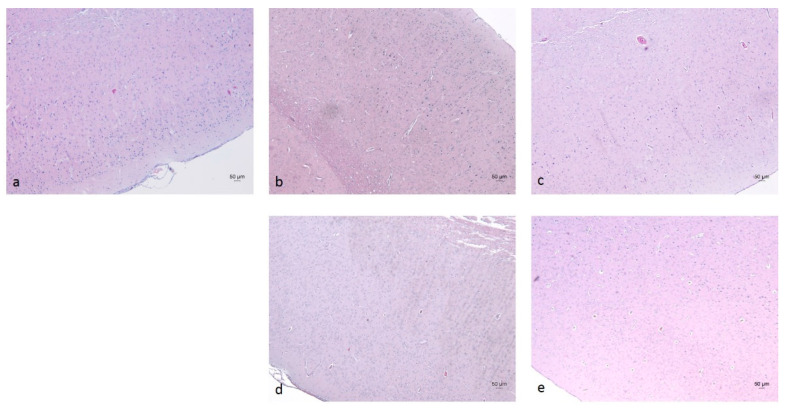
Cerebral cortex of rats from the W-S group (**a**), E-M group (**b**), E-R group (**c**), E-A group (**d**), and E-S group (**e**). Staining: Hematoxylin and Eosin (H&E).

**Figure 7:**
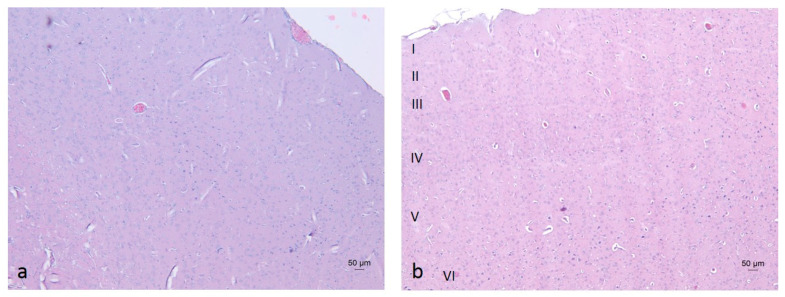
Neocortex of rats from the W-S group (**a**) and E-S group 4 (**b**). Cortical layers: I—Molecular layer (lamina molecularis), II—External granular layer (lamina granularis externa), III—External pyramidal layer (lamina pyramidalis externa), IV—Internal granular layer (lamina granularis interna), V—Internal pyramidal (or ganglionic) layer (lamina pyramidalis interna), VI—Multiform layer (lamina multiformis). Staining: H&E.

In the cerebral cortex (neocortex), the typical vertical arrangement of cortical neurons and the layered organization of glial cells were observed. In rodents, this region represents an isocortical (eulaminate) area characterized by six well-developed layers, including the granular layer IV (Figure 6 and Figure 7) [70,71]. Vascularization appeared unaffected by the daily oral intake of the ethanol–tap water solution (20%, *v*/*v*) at the administered dosage.

**Figure 8:**
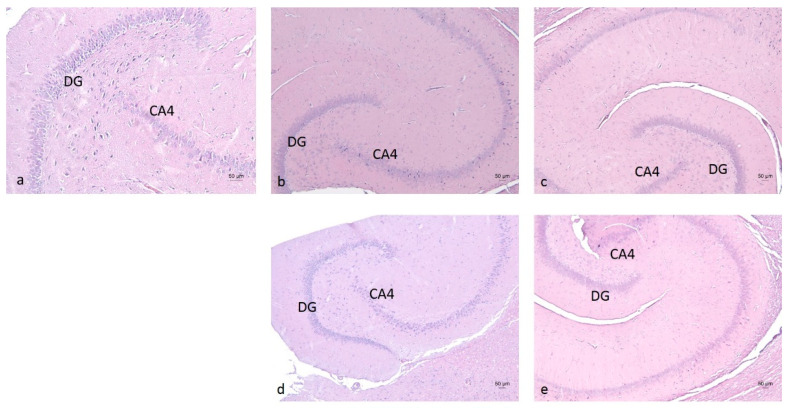
Hippocampus of rats from the W-S group (**a**), E-M group (**b**), E-R group (**c**), E-A group (**d**), and E-S group (**e**). DG—Dentate Gyrus; CA4—Cornu Ammonis subfield 4, located near the hilus’ deep, polymorphic layer of the dentate gyrus. Staining: H&E.

Morphological examination of the hippocampus proper, dentate gyrus, and subiculum—components of the hippocampal formation—revealed normal histological features of nerve cells, accompanied by glial cells, including astrocytes and oligodendrocytes. No signs of nuclear pyknosis or cellular degeneration were observed in any of the four experimental groups (Figure 8).

**Figure 9:**
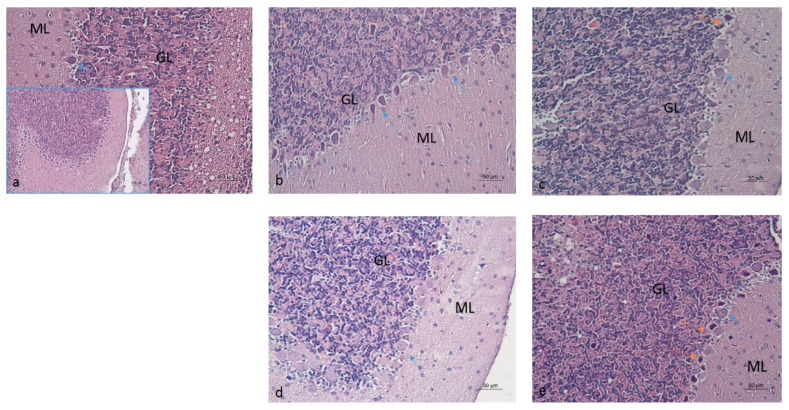
Cerebellar cortex of rats from the W-S group (**a**), E-M group (**b**), E-R group (**c**), E-A group (**d**), and E-S group (**e**). ML—Molecular layer (superficial); GL—Granular layer, containing densely packed small neurons. Blue arrow—Purkinje cell body or dendrite; orange arrow—pyknotic Purkinje cell.

Pathological changes in the cerebellar structures of the E-S group were noted, particularly the degeneration of the cortical Purkinje cell layer adjacent to the granular layer. These changes were characterized by a loss of Purkinje cells, their acidophilic appearance, and abundant nuclear pyknosis (Figure 9).

In contrast, these alterations were not as prominent in the E-M, E-R and E-A groups, although occasional pyknosis was noted in the E-R group, along with altered Purkinje cell phenotypes in the E-R and E-A groups. In the E-R, E-A, and E-S groups, the cell bodies and dendrites of Purkinje cells exhibited reduced acidophilia, suggesting changes in protein content. The cerebellar morphology of the E-M group resembled that of the W-S group. The inner, densely populated granular cell layer remained unaffected in the EGs, maintaining its characteristic organization similar to the W-S group.

Based on the histopathologic examination of cerebellar structures, semi-quantitative scoring of degenerative Purkinje cell alterations was conducted to identify biological differences among the W-S, E-M, E-R, E-A, and E-S groups (Table 2).

These observations suggest that ALA administration had neuroprotective effects on cerebellar structures in the E-A group similar to those achieved with rivastigmine (1.5 mg/kg i.p.), and memantine (5 mg/kg i.p.).

### 2.4. Biochemical Analysis of Brain Tissues

#### 2.4.1. AChE Activity and BDNF Levels

**Figure 10:**
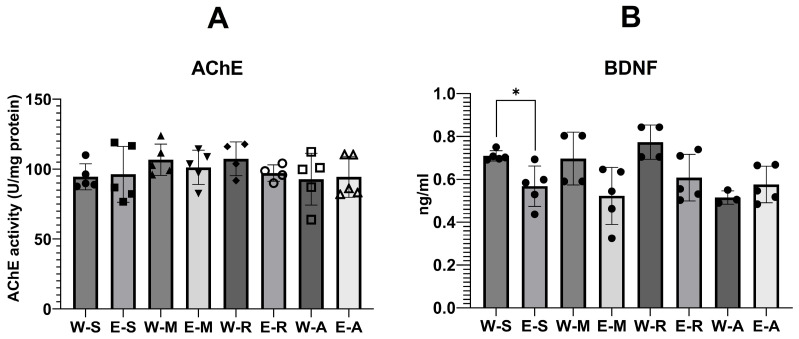
The effects of ALA, rivastigmine, and memantine on brain acetylcholinesterase (AChE) activity (**A**) and brain-derived neurotrophic factor (BDNF) levels (**B**) in the WGs and EGs. Values represent mean ± SEM. (**A**): A one-way ANOVA on Treatment was performed (GraphPad Prism 9). The number of animals per group was *n* = 5 for all groups, except E-R group, where *n* = 4. (**B**): Asterisk above the bars indicates a significant difference in ng/mL between the groups with * *p* < 0.05. One-way ANOVA with fixed factor Treatment was performed (GraphPad Prism 9). The number of animals per group was *n* = 5 for W-S, E-S, E-M, E-R and E-A groups, *n* = 4 W-M and W-R, *n* = 3 for W-A group.

One-way ANOVA indicated no significant differences in AChE activity among groups (Figure 10, F(7,31), ns).

**Figure 11:**
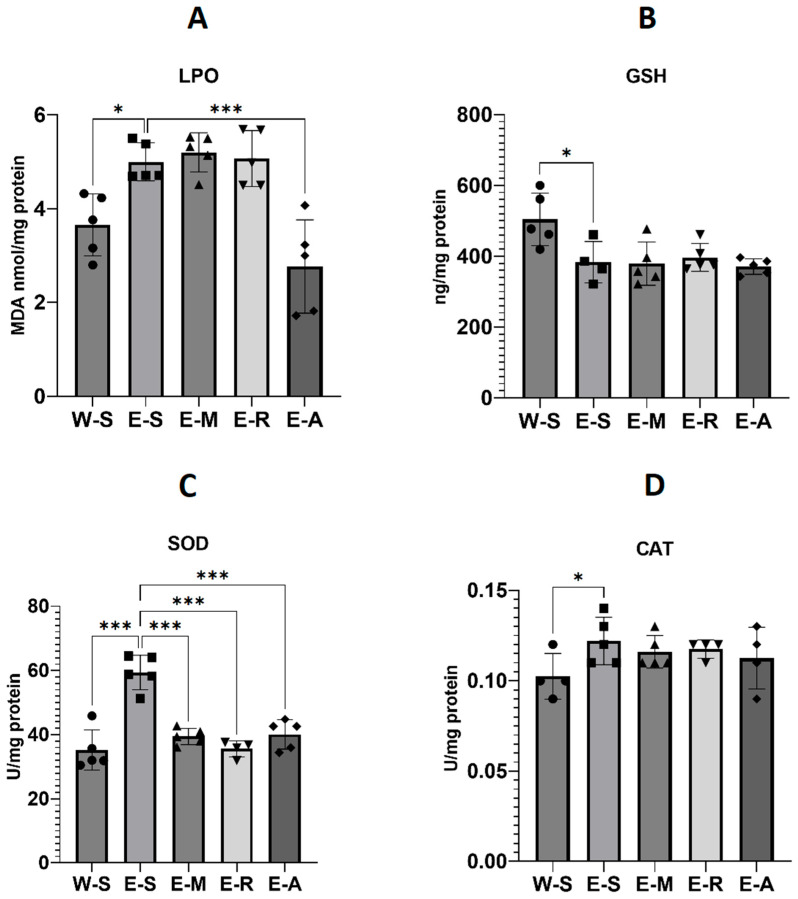
The effects of ALA, rivastigmine, and memantine on brain lipid peroxidation (LPO) (**A**), total glutathione (GSH) (**B**), superoxide dismutase (SOD) (**C**), and catalase (CAT) (**D**) levels in the W-S group and the EGs. Values represent mean ± SEM. Asterisk above the bars indicates a significant difference in ng/mL between the groups with * *p* < 0.05 and *** *p* < 0.001. One-way ANOVA was performed (GraphPad Prism 9). For LPO, the sample size of each group consisted of *n* = 5 animals; For GSH, *n* = 5, except E-S where *n* = 4; SOD, *n* = 5, except E-R where *n* = 4; CAT, W-S *n* = 3, E-S *n* = 5, E-M *n* = 5, E-R *n* = 4, E-A *n* = 4.

BDNF showed a statistically significant effect of the treatment (Figure 11, F(7,28) = 4.16, *p* < 0.01, η^2^ = 0.51). W-S group showed higher mean levels of BDNF (Mean = 0.71 ng/mL ± SD = 0.02 ng/mL) compared with the E-S group (Mean = 0.57 ng/mL ± SD = 0.09 ng/mL, Dunnett’s test, *p* > 0.05). This comprised about 20% decrease in BDNF by the alcohol. Numerically, memantine (W-M, Mean = 0.70 ng/mL ± SD = 0.12 ng/mL vs. E-M, Mean = 0.52 ng/mL ± SD = 0.13 ng/mL) and rivastigmine (W-R, Mean = 0.77 ng/mL ± SD = 0.08 ng/mL vs. E-R, Mean = 0.60 ng/mL ± SD = 0.13 ng/mL, *p* > 0.05). Further, ALA administration numerically slightly increased BDNF levels in E-A group vs. W-A group (W-A, Mean = 0.52 ng/mL ± SD = 0.03 ng/mL vs. E-A, Mean = 0.58 ng/mL ± SD = 0.09 ng/mL, *p* > 0.05).

#### 2.4.2. Oxidative Stress Levels

Lipid peroxidation (LPO, nmol/mg protein) showed a statistically significant effect of treatment (Figure 11, F(4,20) = 13.69, *p* < 0.001, η^2^ = 0.73). Ethanol exposure increased LPO by 35% compared with controls (W-S, Mean = 3.7 ± SD = 0.66 vs. E-S, Mean = 5.0 ± SD = 0.41). ALA markedly decreased LPO to 2.8 ± 0.99, which was 24% lower than W-S and 44% lower than E-S (*p* < 0.001). In contrast, memantine (5.2 ± 0.42, +41% vs. W-S) and rivastigmine (Mean = 5.1 ± SD = 0.59, +38% vs. W-S) did not counteract the ethanol effect.

Total glutathione (GSH, ng/mg protein) was also significantly affected (Figure 11, F(4,22) = 6.19, *p* = 0.002, η^2^ = 0.53). Ethanol reduced GSH relative to controls (W-S, Mean = 504 ± 78 vs. E-S, Mean = 383 ± SD = 58, *p* < 0.05, −24%). None of the treatments restored GSH (E-M, Mean = 379 ± SD = 61, −25% vs. W-S; E-R, 396 ± 39, −21% vs. W-S; E-A, 371 ± 22, −26% vs. W-S; all *p* > 0.05 vs. E-S).

Superoxide dismutase (SOD, U/mg protein) activity revealed a robust treatment effect (Figure 11, F(4,23) = 27.20, *p* < 0.001, η^2^ = 0.83). Ethanol significantly elevated SOD activity compared with controls (W-S, Mean = 35.2 ± SD = 5.06 vs. E-S, Mean = 59.4 ± SD = 5.38, +69%, *p* < 0.001). ALA reduced SOD to Mean = 40.1 ± SD = 4.59 (+14% vs. W-S, −33% vs. E-S, *p* < 0.001), comparable to memantine (39.4 ± 2.56, +12% vs. W-S, −34% vs. E-S) and rivastigmine (35.60 ± 2.52, +1% vs. W-S, −40% vs. E-S).

Catalase (CAT, U/mg protein) showed only a statistical trend toward significance (Figure 11, F(4,16) = 2.57, *p* = 0.08, η^2^ = 0.39). Ethanol increased CAT by 26% compared with controls (W-S, Mean = 0.10 ± SD = 0.01 vs. E-S, 0.12 ± 0.01). Values in drug-treated ethanol groups were moderately elevated vs. W-S but did not differ significantly from E-S (E-M, 0.12 ± 0.01, +20%; E-R, 0.12 ± 0.01, +22%; E-A, 0.11 ± 0.02, +16%)

## 3. Discussion

### 3.1. Step-Through Passive Avoidance Test

Our findings regarding the effects of ethanol on learning and memory in rats in the step-through passive avoidance test are consistent with previous research demonstrating that ethanol and its metabolites can exert long-lasting impairments on cognitive processes. For example, Hasanein et al. (2017) reported that male Wistar rats administered daily 5% *w*/*v* ethanol (2 g/kg, intragastrically) for 30 days exhibited decreased STL, indicating impaired memory [72]. Similarly, Casamenti et al. (1993) reported that male Wistar rats exposed to escalating ethanol concentrations (5% *v*/*v* for 1 week, 10% for 1 week, then 20% thereafter) in drinking water for 3 or 6 months, followed by withdrawal, had significantly lower STL scores at 24 h, with greater deficits after 6 months [45]. A longer experiment by Melis et al. (1996) found that adult male Sprague–Dawley rats—a strain known to consume more alcohol than Wistar rats—receiving gradually increasing ethanol concentrations over 9 months, followed by a two-week withdrawal period, also exhibited significantly reduced STL in step-through passive avoidance tests, indicating lasting memory impairment [73,74]. These results are broadly consistent with ours, though having some differences (e.g., strain, inclusion of a withdrawal period).

### 3.2. AChE Activity

The Melis et al. (1996) study also found a positive correlation between STL and hippocampal acetylcholine (ACh) levels, associating reduced hippocampal ACh release with poorer memory performance. ACh is the main neurotransmitter in cholinergic pathways that underlie attention, learning, and memory, and is hydrolyzed by AChE. Excessive AChE activity lowers cholinergic tone and impairs memory, while its inhibition raises ACh levels and enhances learning and memory. Ethanol’s effect on AChE activity appears to depend on both the specific brain region examined and the pattern of ethanol consumption, with studies reporting decreases [75,76,77,78,79,80,81], increases [82,83,84], or no change [80,83,85,86,87,88]. Pereira et al. (1998) fed male Fisher rats 20% ethanol in an 8.75% sucrose solution as the sole drinking fluid for 32 weeks, followed by withdrawal, and found no change in cortical AChE activity [87]. A later study by Pires et al. (2001) in adult male Wistar rats given 20% *v*/*v* ethanol as their sole drinking fluid for 4 months also found no change [80]. Ruano et al. (2000) found no change in the activity of the membrane-bound form of AChE in the forebrain, cerebellum, or brain stem of adult male Wistar rats exposed to 20% *v*/*v* ethanol for 10 months, but did find an increase in the soluble form in the brain stem [88].

These findings are consistent with ours, although it should be noted that our analyses were performed in whole-brain homogenates, which may have masked region-specific effects. Lieberthal et al. (1980) assessed AChE activity in whole-brain samples in vitro and found that ethanol did not inhibit the enzyme to any appreciable extent (<5%) [75]. In contrast, Guerri and Grisolía (1983) observed an increase in AChE activity in whole-brain homogenates; however, their use of a liquid ethanol-containing diet likely resulted in sustained elevated blood alcohol levels, differing from the variable intake associated with an *ad libitum* ethanol–tap water solution, as used in our study [83].

No data were found regarding the effects of ALA on AChE activity in animal models of ethanol administration. Our previous data in other Wistar rat models showed reduced AChE activity in the hippocampus and prefrontal cortex after short-term treatment, and in the hippocampus after long-term treatment with higher doses [48,89]. We therefore suggested that ALA could potentially counteract ethanol-induced increases in AChE activity [82,83,84]. However, findings on brain AChE activity from other experimental contexts have been mixed, with some studies reporting an increase [90,91,92] and others a decrease [46,93,94] following ALA administration. Our present data did not show significant changes in brain AChE activity in the ALA-treated ethanol group (E-A group). Thus, it is possible that this mechanism may not contribute to the improved learning and memory observed in our study. However, given that ethanol-induced oxidative stress may suppress AChE activity, and considering the significant antioxidant potential of ALA alongside its documented effects on AChE activity in other experimental models, future studies should investigate AChE activity in specific brain regions [95,96].

Rivastigmine did not appear to inhibit AChE activity, yet produced a clear preventive effect in the step-through passive avoidance test compared with the untreated E-S group. Thus, the results may reflect a methodological limitation of measuring AChE activity in whole-brain homogenates rather than within specific brain regions. [81]. In contrast, memantine, an NMDA receptor antagonist, is not expected to inhibit AChE activity—a finding confirmed by our study [97].

### 3.3. BDNF Levels

ARBD has been linked to altered neurotrophic activity, particularly reduced BDNF levels, which can impair neuronal plasticity and cognitive function [35]. BDNF, a key molecular mediator of memory, supports synaptogenesis and synaptic remodeling [98]. In our study, BDNF levels were significantly decreased in the E-S groups compared with the W-S groups, consistent with previous findings [35,99,100,101,102,103,104]. However, no data were found regarding the effects of ALA on BDNF levels in animal models of ethanol administration. In other experimental contexts in rats, ALA has been shown to elevate BDNF levels; however, these effects were observed at substantially higher doses (100–200 mg/kg) than that used in our study (25 mg/kg i.p.) [105,106,107]. We did not find significant changes in neurotrophic activity, as measured by BDNF levels, in the ALA-treated ethanol group (E-A). These initial findings in this animal model motivate us to investigate whether higher doses of ALA can counteract the BDNF deficit, ideally by examining specific brain regions rather than whole-brain homogenates. Similarly, the reference compounds rivastigmine and memantine also did not alter BDNF levels in this model.

### 3.4. Oxidative Stress Parameters

It is well established that ethanol consumption increases the production of reactive oxygen species (ROS) and thereby oxidative damage across various tissues [50]. Similarly, in our experiment, we found that ethanol consumption significantly increased LPO, indicating elevated oxidative damage in brain tissue. The activities of the antioxidant enzymes SOD and CAT were also elevated, likely reflecting a compensatory response to heightened oxidative stress; notably, CAT activity has been reported to rise with chronic alcohol consumption [50,108]. At the same time, levels of GSH, a critical non-enzymatic antioxidant, were significantly decreased in all EGs, suggesting its depletion in response to sustained oxidative challenge and consistent with previous reports of GSH reduction following chronic alcohol intake [50,109,110,111].

The pleiotropic antioxidant properties of ALA are well documented (Table 1). Administration of ALA following ethanol exposure significantly reduced LPO—unlike memantine and rivastigmine—indicating a mitigation of oxidative damage. ALA also decreased the levels of SOD—similar to memantine and rivastigmine—which may reflect a diminished need for antioxidant defense as oxidative stress is alleviated. In a previous study, we demonstrated a similarly strong antioxidant effect of ALA in a rat model of scopolamine-induced dementia [37], and the present reduction in LPO aligns with those findings. However, in the current study, CAT and GSH levels remained largely unchanged by ALA. A double-blind, randomized clinical trial in 63 hemodialysis patients found that daily administration of 600 mg ALA for eight weeks also did not increase CAT activity [112]. In another study, two weeks of 0.2% (*w*/*w*) ALA given together with an AIN-93M diet failed to alter GSH levels in young (4–6 month) Fischer 344 male rats—an age similar to that of our animals—but significantly increased GSH in older (26–28 month) rats, indicating an age-dependent effect [113]. Thus, the pattern we observed could be due to ALA shifting the oxidative balance by directly reducing reactive species and thus the need for endogenous antioxidant enzyme upregulation, rather than by replenishing GSH stores. Additionally, the use of whole-brain homogenates may have masked region-specific or subcellular (mitochondrial vs. cytosolic) alterations, and a longer dosing period may have been required. It is likely that the near-equivalent memory-enhancing effects of ALA to those of memantine and rivastigmine at both 24 h and 8 days in the step-through passive avoidance test are due to its antioxidant properties demonstrated in our study and in the literature (Table 1).

### 3.5. Body Weight, Brain Weight and Relative Brain Weight

Data on the effects of chronic ethanol exposure on brain weight in adult rats remains limited. Studies using adolescent rat models have reported ethanol-induced reductions in brain weight [95,114,115]. However, the immature brain is considered significantly more vulnerable to the damaging effects of ethanol compared to the adult brain, and underlying mechanisms of ARBD are known to differ between the two. In the present study, adult male rats—older than 6 weeks—were used, an age by which over 90% of the adult brain weight has already been attained [116]. Additionally, male rats are generally considered less susceptible to ethanol-induced brain damage, which may be due to sex-related dimorphisms in brain development [117].

In a study by Yalcin et al. (2017) using adult male Long-Evans rats fed an isocaloric liquid diet containing 26% ethanol by caloric content (approximating 6% *v*/*v*) for 3 or 8 weeks, in combination with a binge-like pattern of intraperitoneal ethanol injections (2 g/kg, three times per week) during the final 2 weeks, reported no significant changes in mean brain weight [118]. In contrast, a more recent study by Tong et al. (2025) investigated adult male and female Long-Evans rats fed a Lieber–DeCarli isocaloric liquid diet containing 36% ethanol by caloric content (approximating 8% *v*/*v*) for periods ranging from 1 day to 8 weeks, following a four-day adaptation phase. While no significant alterations in mean brain weight were observed during the first two weeks, significant ethanol-induced reductions emerged at 4, 6, and 8 weeks of exposure [116]. However, it is important to note that the animals were maintained on a Lieber-DeCarli isocaloric liquid diet and consistently consumed ≥90% of it, leading to sustained and elevated blood alcohol concentrations. By contrast, the ethanol–tap water solution administered *ad libitum* in our study likely resulted in more variable ethanol intake and fluctuating blood alcohol levels leading to inconsistent patterns of brain damage. Additionally, Ruano et al. (2000) administered a 20% (*v*/*v*) ethanol solution as the sole source of liquid to adult male Wistar rats for 10 months—a regimen similar to ours but of much longer duration—and also reported no change in the weight of the brain areas examined (forebrain, cerebellum, and brain stem) [88]. Therefore, our findings are likely attributable to the animals’ age, as well as the *ad libitum* ethanol intake regimen.

The general trend toward increased relative brain weight in the EGs likely reflects ethanol-induced reductions in body weight over the 15-week period—a denominator effect and limitation of relative organ weight measurements—rather than a true increase in brain mass, consistent with previous reports [45,73,80,88,119]. The body weight reductions observed during the final week (injection period) per se in the E-R and W-A groups may be related to the capacity of ALA [120,121,122,123,124,125] and rivastigmine [126] to suppress appetite and promote weight loss in certain contexts. Acetylcholinesterase inhibitors such as rivastigmine can also induce nausea in both animals and humans [127,128], further limiting weight gain. Thus, the lack of significant weight recovery could reflect other pharmacological properties of these compounds rather than a lack of therapeutic efficacy. It is also possible that brain edema could have developed during the course of ethanol exposure. Research using rat models and cultures has demonstrated that ethanol can induce brain edema by upregulating aquaporin-4 (AQP4) channels, as well as increasing inflammation and oxidative stress [129,130,131,132,133], which may account for the present findings. Importantly, the primary neuroprotective outcomes—behavioral performance, oxidative stress markers, and cerebellar histology—demonstrated clear benefits with ALA treatment.

It is important to note that the cumulative weight gain in our study was modest compared to that of growth-phase animals, consistent with the rats being in the young adult stage (180–200 g at baseline), when growth typically stabilizes [134,135]. Chronic ethanol consumption is known to suppress appetite and reduce weight gain, which was evident across all ethanol-consuming groups [136,137]. The marked weight changes observed during the final week of injections likely reflect temporary influences such as hydration status and fluid intake patterns, factors known to affect short-term weight measurements in rodents [138]. Importantly, relative differences among groups remained consistent, and both brain weight and biochemical outcomes showed internal coherence, suggesting that this variability did not affect the interpretation of neurobiological findings. Similar fluctuations in weight trajectories during prolonged ethanol exposure have also been reported in other rodent studies [88].

### 3.6. Histopathological Analysis

Chronic alcohol consumption in humans has been associated with Purkinje cell loss [139]. Ethanol-induced degenerative changes in the cortical Purkinje cell layer of adult rats have also been reported [140,141,142], although much of the existing research has focused on developmental (postnatal and adolescent) models [143,144,145]. In our study, ALA reduced oxidative stress markers, which—along with other mechanisms (Table 1)—may underlie its observed neuroprotective effects [142,146,147]. The neuroprotective effect was similar to that achieved with rivastigmine and memantine.

### 3.7. Rotarod Test

The pathological changes were observed in the cerebellar structures of the E-S group and did not appear sufficient to cause motor coordination impairments detectable by the rotarod performance test. The effect of ethanol on motor coordination appears to vary depending on age, exposure pattern, and test design. Most studies employing the rotarod performance test in the context of chronic or binge ethanol exposure have been conducted using developmental—postnatal or adolescent—rather than adult rat models, where ethanol has been shown to impair motor coordination, as evidenced by reduced rotarod performance and corresponding histopathological findings. In these models, the developing brain may exhibit greater vulnerability to ethanol-induced damage compared to the adult brain [117,143,148,149,150,151,152].

In a study by Dulman et al. (2021), adult male Sprague–Dawley rats were maintained on a liquid diet in which ethanol concentration increased from 1.8% to 9% over 7 days and was then sustained at 9% for 15 days. Rotarod testing was conducted during ethanol exposure using an apparatus that started at 5 rpm and accelerated to 20 rpm over 180 s (cut-off), with latency to fall recorded for each trial. Ataxia was observed during chronic ethanol treatment [153]. However, a liquid-diet approach is expected to yield higher and less variable blood ethanol concentrations (BECs) than those produced in our *ad libitum* model, particularly in Sprague–Dawley rats, which generally consume more ethanol than Wistar rats [74]. In addition, our rotarod protocol was less demanding, using a fixed slow speed of 7 rpm rather than the higher, accelerating speeds up to 20 rpm. We also measured total fall count, which is a lower-resolution metric than latency to fall.

A study more similar to ours, by Wang et al. (2020), provided adult male Sprague–Dawley rats with *ad libitum* access to ethanol as their sole drinking fluid for 60 days, gradually increasing concentration from 6% to 20% by day 15 (1%/day). The rats were trained on a fixed-speed rotarod at 20 rpm from day 54 to day 58 and tested on day 59. Latency to fall was recorded with a 300 s cut-off. This protocol was more sensitive than ours: 20 rpm vs. 7 rpm; latency vs. fall count; 300 s vs. 180 s cut-off. Nevertheless, no significant difference in latency to fall was observed between ethanol and control groups [149]. These findings, together with ours, are most likely explained by the development of tolerance to ethanol’s motor-impairing effects [154]. Similarly, Uzbay et al. (1995) found that in adult male Wistar rats given 7.2% ethanol *ad libitum* for 15 days, rotarod performance was impaired on days 2 and 4 but returned to baseline by day 7 and remained unchanged through day 15, while impairment persisted on the accelerod [155].

Taken together, these results suggest that the absence of ataxia in our study most likely reflects the combined influence of age, a less sensitive rotarod protocol (slow, constant speed) and low/variable BECs in the context of tolerance. Consequently, there may have been little to no detectable dysfunction for ALA to influence. The same could be said about rivastigmine, although one study using doses of 0.5 and 1.0 mg/kg [63], and another using 1.5 mg/kg [156]—the latter matching our own—also failed to produce significant improvements in rat performance on the rotarod apparatus. The increased number of falls observed in the W-M and E-M groups at 1 h is likely due to memantine’s known capacity to disrupt locomotor behavior, even at doses as low as 5 mg/kg—the dose employed in this study [156,157].

### 3.8. Summary and Limitations

In summary, our findings in this comparative pilot study demonstrate that ALA exerted robust neuroprotective effects in an early-stage ARBD rat model, showing a memory enhancement in the step-through passive avoidance test and neuroprotective effects on cerebellar structures similar to those of the reference drugs memantine and rivastigmine. These benefits appear most closely linked to its potent antioxidant properties, evidenced by significant reductions in LPO—unlike the reference drugs—and SOD activity, similar to the reference drugs. Notably, to the best of the authors’ knowledge, this is the first comparative study to evaluate ALA alongside memantine and rivastigmine in an early-stage ARBD rat model, and the first to assess the investigated parameters in this context.

However, several limitations of the present study should be acknowledged. First, the experiments employed single, relatively small doses over a short treatment period, which limits conclusions regarding dose–response relationships and the potential for optimal therapeutic dosing. Future studies should therefore examine multiple concentrations and treatment regimens.

Second, the rotarod test and biochemical analyses provided relatively low resolution. In particular, the latter were performed using whole-brain homogenates, which may have masked region-specific effects of ethanol and the tested compounds. Region-targeted analyses would be valuable for clarifying the mechanisms underlying the observed changes and for determining whether particular neural circuits are more responsive to the effects of ALA.

Third, ethanol exposure in this model was provided via *ad libitum* intake. While this approach mimics voluntary consumption, it introduces variability in individual intake levels, potentially contributing to within-group heterogeneity. Future studies may benefit from combining voluntary drinking paradigms with controlled administration approaches.

Fourth, an important limitation of the present study concerns the restricted sample size. The number of animals per group was determined a priori using the statistical software G*Power 3.1, which indicated that with *n* = 6, α = 0.05, and power (1–β) = 0.80, the design was sensitive to detect medium-to-large effect sizes (Cohen’s f ≥ 0.40). However, due to occasional losses, several groups were reduced to *n* = 3–4. For these groups, the corresponding findings are presented only as numerical trends or complementary additional effects, without claims of statistical significance. The limitation in sample size was mandated by the Institutional Ethical Committee, in accordance with the principles of the 3Rs (Replacement, Reduction, Refinement). The study was therefore conducted under the requirement to use the minimum number of animals necessary to achieve valid conclusions while ensuring animal welfare. This approach is consistent with established guidelines emphasizing the need to reduce animal use through better experimental design [158,159,160].

Finally, although the model provides valuable insights into ethanol-induced memory impairment and oxidative stress, as well as their modulation by ALA, the translational relevance to human ARBD should be interpreted with caution. Differences in metabolism, dosing, chronicity of alcohol exposure, and the complexity of comorbid conditions in humans may limit direct applicability. Longitudinal studies in animal models that incorporate varying ages, sex differences, and comorbidities, as well as clinical investigations, will be important next steps for establishing the therapeutic potential of ALA in ARBD.

## 4. Materials and Methods

### 4.1. Experimental Substances

Alpha-lipoic acid was administered as Thioctacid^®^ 600 T 600 mg/24 mL solution for injection (MEDA Phanna GmbH & Co., KG, Homburg, Germany). Rivastigmine was administered as rivastigmine tartrate (PHR1867-1G, Sigma-Aldrich, Germany) and memantine as memantine hydrochloride (M9292-100MG, Sigma-Aldrich, Darmstadt, Germany). The ethanol–tap water solution (20%, *v*/*v*) was prepared by using Spiritus Aethylicus (95%, *v*/*v*) (GALEN Pharma, Kalugerovo, Bulgaria) and tap water.

### 4.2. Experimental Animals

All experimental procedures were conducted on adult male Wistar rats. Using adult animals avoided confounding effects of ongoing brain maturation in juveniles. Restricting the study to males minimized variability related to estrous cycle–dependent hormonal fluctuations in females and increased translational relevance, as ARBD prevalence is higher in men than in women [6,161], although adult female rats are known to consume more alcohol than males [162].

The animals averaged 180–200 g upon experiment onset, bred in the Laboratory Animal Resource Center of Bulgarian Academy of Sciences’ Institute of Neurobiology (Slivnitsa, Bulgaria). The animals were transported and housed first in a laboratory in the Department of Pharmacology and Toxicology of Medical University of Sofia’ Medical Faculty (Sofia, Bulgaria) and then in a laboratory in the Institute of Neurobiology of Bulgarian Academy of Sciences (Sofia, Bulgaria). After transportation, the animals were allowed to acclimate to their environment before the start of experiments. The laboratory environments did not differ significantly in their 12 h light/dark cycles, temperature, humidity, and ventilation. In both laboratories, the animals were group-housed (*n* = 6–7) in plastic cages and allowed to habituate to their environments. They were provided continuous access to food (pelleted diet), and either tap water or an ethanol–tap water solution (20%, *v*/*v*) per their group. Experimental procedures were conducted in view of national and international regulations and the institutional guidelines of Medical University of Sofia and Bulgarian Academy of Sciences.

### 4.3. Experimental Design

Adult male Wistar rats (*n* = 48) were randomly assigned to 8 experimental groups of 6 animals each using simple randomization. The lead researcher was blinded to group allocation during this process.

An experimental rat model of early-stage ARBD was generated in 4 groups by providing them with an ethanol–tap water solution (20%, *v*/*v*) *ad libitum* as their sole drinking fluid. The sole drinking fluid for the other 4 groups was tap water. After drinking for 14 weeks, all 8 groups were injected each day for 7 days with either of the following drugs: ALA, rivastigmine, memantine, or 0.9% NaCl. The ethanol and drug doses were chosen based on prior experiments [29,48,163] and published literature [156,164,165,166]. Each group was injected with only one drug. The animals receiving the ethanol–tap water solution continued on it throughout the injection period, bringing the total duration of ethanol exposure to 15 weeks.

The following water-drinking groups (WGs) drank only tap water and were injected as described:

- Water–Saline group (W-S), injected with 0.9% NaCl (0.5 mL/100 g i.p.).
- Water–ALA group (W-A) injected with ALA (30 mg/kg i.p.).
- Water–Rivastigmine group (W-R), injected with rivastigmine (1.5 mg/kg i.p.).
- Water–Memantine group (W-M), injected with memantine (5 mg/kg i.p.).

The following ethanol-drinking groups (EGs) drank only an ethanol–tap water solution (20%, *v*/*v*) and were injected as described:

- Ethanol–Saline group (E-S), injected with 0.9% NaCl (0.5 mL/100 g i.p.).
- Ethanol–ALA group (E-A), injected with ALA (30 mg/kg i.p.).
- Ethanol–Rivastigmine group (E-R), injected with rivastigmine (1.5 mg/kg i.p.).
- Ethanol–Memantine group (E-M), injected with memantine (5 mg/kg i.p.).

Behavioral phenotyping was done for 8 days—in conjunction with the 7-day injection period—using a step-through passive avoidance test and a rotarod performance test. On the final day of testing, the rats were euthanized by CO_2_ inhalation. They were then decapitated and their brains were quickly removed, weighed, and stored for analysis. Histopathological examination of the cerebral white and grey matter, hippocampal formation, and cerebellum was performed in the W-S group and EGs. Whole-brain measurements of AChE and BDNF were obtained in the WGs and EGs, while oxidative stress biomarkers—LPO, GSH, SOD, and CAT—were assessed in the W-S group and EGs. The data were subsequently subjected to statistical analysis. Experimental procedures and statistical analyses were conducted by different investigators.

**Figure 12:**
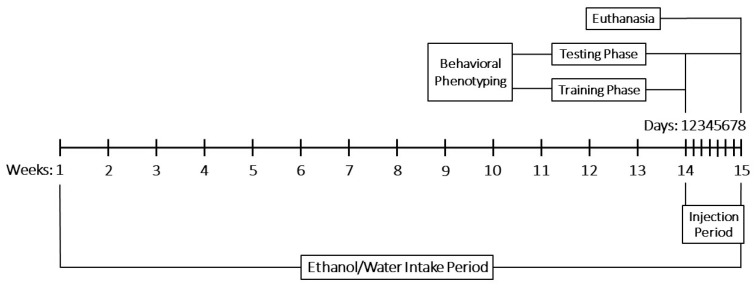
Experimental timeline.

An overview of the experimental timeline is shown in Figure 12.

#### 4.3.1. Behavioral Phenotyping

Behavioral data acquisition was carried out using a step-through passive avoidance test to evaluate changes in learning and memory and a rotarod performance test to evaluate changes in motor coordination.

- Step-through Passive Avoidance Test [167]

The Step-through Passive Avoidance Test is a fear-aggravated test used to evaluate the effect of novel chemical entities on learning and memory in rodent models of central nervous system disorders. In this neuropharmacological task, rodents learn to avoid an environment where an aversive stimulus (e.g., a foot shock) was previously delivered.

Its two-chambered testing apparatus is divided into a lit chamber and a dark chamber, with a gate in between. The floor of the dark chamber has a steel grid through which a mild electric foot shock can be given. If done so, the rodents will learn to associate certain properties of the dark chamber with the foot shock. Rodents with normal learning and memory will then avoid entering the dark chamber. This is measured by recording the latency to cross through the gate between the chambers.

The experimental procedure was two-phased: training and testing. The training phase was on the same day as the injection period, preceding it. During the training phase, each rat was placed in the lit chamber, and after a one-minute habituation period, the gate was opened. That allowed the rat to enter the dark chamber, whereafter it received a one-time electric foot shock (0.7 mA, 3 s). The time it took the rat to enter the dark chamber with all four paws was written down as initial latency (IL). The training phase was followed by the testing phase. The testing phase was during the injection period. One hour, twenty-four hours, and eight days, respectively, after the first ALA, rivastigmine, memantine, or 0.9% NaCl injection, each rat was placed again in the lit chamber and allowed to enter the dark chamber but without receiving a foot shock. The time it took the rat to enter the dark chamber with all four paws was written down as STL. To reliably assess memory changes, a standard cut-off time of 180 s was applied for IL and STL.

- Rotarod Performance Test [168]

The rotarod performance test is a fear-aggravated test used to evaluate the effect of novel chemical entities on motor coordination and balance in rodent models of central nervous system disorders. In this neuropharmacological task, the rodents’ natural fear of falling is used as motivation.

Its testing apparatus has a rotating PVC rod with a non-slip surface divided by spacer disks. The rats are placed between the spacer disks and try to balance at a pre-specified speed of rotation.

The experimental procedure was two-phased: training and testing. The training phase was on the day of the injection period, preceding it. During the training phase the rats were placed on the rotating rod (7 rpm) to learn to balance on it. If they fell, they were placed back on until the cut-off time of 180 s had elapsed. The training phase was followed by the testing phase. The testing phase was during the injection period. One hour, twenty-four hours, and eight days, respectively, after the first ALA, rivastigmine, memantine, or 0.9% NaCl injection, each rat was placed again on the rotating rod (7 rpm). If they fell, they were placed back on until the cut-off time of 180 s had elapsed. The number of falls/min was recorded.

#### 4.3.2. Body Weight, Brain Weight and Relative Brain Weight Measurement

Body weight was measured with a mechanical balance. Brain weight was measured with an electronic balance. Subsequently, brain weight relative to body weight was calculated by using the formula:

$$\frac{BrainWeight}{BodyWeight}\times 100$$

#### 4.3.3. Histopathological Analysis of Brain Tissues

Brains from randomly selected representatives of each group were histologically analyzed to examine and compare their cytoarchitecture, aiming to identify morphological features that correlate with behavioral and biochemical parameters. The primary areas of interest included the cerebral white and gray matter, the hippocampal formation, and the cerebellum.

Whole brains were placed in 10% formaldehyde solution for 24 h and then in a new formaldehyde solution for another 24 h before being dehydrated with 70%, 90%, and 100% ethanol. Brain tissue was standardly cleaned in xylene and embedded in paraffin. Sections were cut at 5 µm thickness, mounted, and stained with a routine H&E technique. Examination of slides was done under a light microscope (Leica DM 5000B, Wetzlar, Germany).

#### 4.3.4. Biochemical Analysis of Brain Tissues

- AChE Levels [169]

The Ellman method was used for biochemical analysis of brain AChE levels. It is a spectrophotometry-based assay in which thiocholine, produced by AChE, reacts with 5,5′-Dithiobis (2-nitrobenzoic acid) (Ellman’s reagent) to form a yellow-colored product, proportional to the AChE activity present.

Whole-brain AChE activity was determined. Brains were frozen at −25 °C, cut into smaller fragments and weighed. The fragments were mixed together with phosphate-buffer solution (PBS) (0.01 mol/L, pH = 7.4) and homogenized (tissue weight (g): PBS (mL), volume = 1:9) with a Potter-Elvehjem Glass Homogeniser with a Teflon pestle. The homogenate was then centrifuged at 5000× *g* for 5 min, and the resultant supernatant was used to determine AChE activity. All procedures were kept under temperature control, between 0 °C and +4 °C. 100 mcl of supernatant were incubated with the Ellman reagent: 2.9 mL of 0.1 M phosphate buffer (pH = 8), 100 mcl of 0.1 M Ellman reagent, and 20 mcl of 0.075 M freshly prepared acetylthiocholine iodide. 500 mcl of the reaction mixture was then analyzed with a semi-automatic biochemistry analyzer (Shenzhen Mindray Bio-Medical Electronics Co., Ltd., Shenzhen, China) and reaction kinetics were monitored for 3 min at 405 nm. Probe protein concentration was measured using the colorimetric Biuret method.

- BDNF Levels

BDNF levels were measured in whole-brains by the use of a commercially available ELISA Sandwich Kit (MyBioSource Inc., San Diego, CA, USA, Catalog No MBS457896). The assay was carried out according to instructions; 100 µL of BDNF Standard (0.156–10 ng/mL) and samples were added to the 96-well BDNF pre-coated plate. The plate was then covered and incubated at 37 °C for 90 min, after which the liquid from each well was removed. After all the liquid was removed 100 µL of Detection Solution A was added to the wells, followed by an incubation at 37 °C for 45 min and subsequent wash of the wells with a Wash Buffer repeated 3 times for 1–2 min. Next to each well was added 100 µL of Detection Solution B and the plate was incubation at 37 °C for 45 min. Afterwards the plate was washed with Wash Buffer 5 times and TMB Substrate Solution was added and incubated for 15–25 min. The addition of 3,3′,5,5′-tetramethylbenzidine (TMB) started the color reaction, which was stopped by a Stop Solution 10 min later, followed by the instant record of the absorbance at 45 nm (MR-96A microplate reader, Shenzhen, China). Standards and samples were run and BDNF levels were calculated by the use of a standard curve.

- Oxidative Stress Levels

Rat whole-brain tissues were stored in a freezer at −80 °C until the beginning of biochemical testing. On the day of testing, the samples were defrosted and homogenized within a 0.15 M potassium phosphate buffer and centrifuged for 10 min at 3000 rpm. The obtained ‘postnuclear’ fraction was used to determine LPO and GSH levels. Part of this postnuclear fraction was recentrifuged for 20 min at 12,000 rpm. The resultant postmitochondrial supernatant was used to measure antioxidant enzyme activities—SOD and CAT. All procedures were done at temperature control, between 0 °C and +4 °C.

Protein content was determined by the method of Lowry et al. (1951) [170] by using a calibration curve obtained with bovine serum albumin (Pentex USA).

All oxidative stress biomarkers were measured spectrophotometrically with commercially available kits (Sigma-Aldrich) and by strictly following manufacturer’s instructions:

LPO was measured with an MDA Assay Kit (MAK085, Sigma-Aldrich) and expressed in nmoles malondialdehyde (MDA)/mg protein with a molar extinction coefficient of 1.56 × 10^5^ M^−1^cm^−1^.

GSH content was measured using a Glutathione Assay Kit (CS0260, Sigma-Aldrich) and expressed in ng/mg protein.

SOD activity was measured with a SOD Assay Kit-WST (19160, Sigma-Aldrich) and expressed in U/mg protein.

CAT activity was measured with a Catalase Activity Assay Kit (CAT 100, Sigma-Aldrich) and expressed in U/mg protein.

#### 4.3.5. Statistical Analysis

The statistical analysis was performed using GraphPad Prism software. The following statistical methods were employed in processing the data. Descriptive statistics, including mean, median, standard deviation, standard error of mean and percentages. Shapiro–Wilk normality test—applied to assess whether the data followed a normal distribution. A non-parametric Kruskal–Wallis test method was used for comparing more than two independent variables when the assumptions of normality are not met. One-way and two-way analysis of variance ANOVA was employed to compare means across three or more independent groups, provided that the data followed a normal distribution and exhibited homogeneity of variance. Group contrasts were analyzed using Dunnett’s test. Repeated measures ANOVA—used to evaluate dependent samples over time or under different experimental conditions, with correction methods applied to control for type I error, including the False Discovery Rate (FDR) and Bonferroni correction. Mixed ANOVA—applied to assess the effects of treatment with different substances on behavioral responses over time, accounting for both within-subject and between-subject factors. Three-factor mixed linear model (MixedLM)—used to evaluate the effects of chronic administration (water or ethanol), acute drug administration (saline, memantine, rivastigmine, or alpha-lipoic acid), and time after training (1 h, 24 h, 8 days) on STL in the Step-Through Passive Avoidance test. The model included all two-way and three-way interactions and incorporated a random intercept for each animal to account for repeated measures. Implemented in R, using the knitr, car, emmeans, and multcomplibrary libraries. Three-factor factorial design with Time, Drug, and Alcohol as fixed effects—used to analyze rotarod performance. Individual animals were included as a blocking factor to account for repeated measures. Due to the unbalanced nature of the dataset, Type III sums of squares were used to assess each main effect and two-way interaction after controlling for all other terms in the model. The statistical model included all main effects and two-way interactions (Time × Drug, Time × Alcohol, Drug × Alcohol), along with a blocking term for animal ID.

The number of animals was restricted in accordance with the decision of the Institutional Ethical Committee, which follows the principles of the 3Rs (Replacement, Reduction, Refinement). The study design was specifically adapted to use the minimum number of animals required for valid analysis while maintaining animal welfare standards. The sample size was determined a priori using the statistical software G*Power 3.1. For *n* = 6 animals per group, with α = 0.05 and power (1–β) = 0.80, the calculation indicated sufficient sensitivity to detect medium-to-large effect sizes (Cohen’s f ≥ 0.40) in mixed-design ANOVAs and mixed linear models. Due to occasional losses, some experimental groups had fewer than 6 animals (*n* = 3–4). Data from these smaller groups are reported for completeness; however, given the reduced sample size, the corresponding effects consider them as complementary observations only.

These statistical approaches enabled a robust evaluation of the data and supported the reliability of the conclusions drawn from the analysis.

## 5. Conclusions

ALA (25 mg/kg i.p. for 7 days) administered to an early-stage ARBD rat model, improved ethanol-induced memory decline in the step-through passive avoidance test and exerted neuroprotective effects on cerebellar structures—both similar to the reference drugs memantine and rivastigmine. ALA’s protective effects could be linked to its potent antioxidant capacity, as evidenced by significant reduction in LPO—unlike the reference drugs—and reduction in SOD activity, similar to the reference drugs. Notably, to the best of the authors’ knowledge, this is the first comparative study to evaluate ALA alongside memantine and rivastigmine in an early-stage ARBD rat model, and the first to assess the investigated parameters in this context. However, several limitations should be acknowledged, including the use of single, relatively low drug doses over a short treatment period, variability in individual *ad libitum* ethanol intake, reliance on whole-brain homogenates for biochemical analyses, and a restricted sample size. Nonetheless, the findings highlight ALA’s potential as a therapeutic candidate for mitigating ethanol-induced cognitive impairment and oxidative brain injury.

## Figures and Tables

**Table 1:** Possible mechanisms of action of ethanol and alpha-lipoic acid. The following can vary in terms of experimental model, anatomical region, ethanol regimen, etc. Note: ↑ = increase; ↓ = decrease; → = leads to/causes. [30,31,32,33,34,35,36,37,38,39,40,41,42,43,44,45,46,47,48,49,50,51,52,53,54].

MECHANISM	ETHANOL	ALPHA LIPOIC ACID
Nuclear factor kappa B (NF-κB) transcription → oxidative stress-inducing enzymes and pro-inflammatory cytokines synthesis	↑ NF-κB transcription → ↑ the activity of oxidative stress-inducing enzymes and pro-inflammatory cytokines	↓ NF-κB transcription → ↓ the activity of oxidative stress-inducing enzymes and pro-inflammatory cytokines
Phosphorylated cAMP Response Element-Binding Protein (pCREB) transcription	↓ pCREB transcription → neurons are more vulnerable to oxidative stress-induced damage	↑ pCREB transcription → neurons are less vulnerable to oxidative stress-induced damage
Tumor necrosis factor alpha (TNFα) levels	↑	↓
Lipid peroxidation (LPO), nicotinamide adenine dinucleotide phosphate oxidase (NOX), superoxide dismutase (SOD), and catalase (CAT) activities → ↑ formation of reactive oxygen species (ROS)	↑	↓
Phospholipase A2 (PLA2) and cyclooxygenase-2 (COX-2) activities → ↑ formation of pro-inflammatory prostaglandins	↑	↓
Inducible nitric oxide synthase (iNOS) activity	↑ → ↑ levels of nitric oxide (NO) free radicals	↓ → ↓ levels of NO free radicals
Toll-like receptor 2 and 4 (TLR2/TLR4) neuromodulation pathways	↑ cytokine induction by TLR2 and TLR4 ligands	↓ TLR2 and TLR4 expression
Total glutathione (GSH) levels	↓ the glutathione/glutathione disulfide (GSH/GSSG) ratio → ↓ antioxidant protection	↑ GSH levels → ↑antioxidant protection
Brain-derived neurotrophic factor (BDNF) levels	↓ → ↓ neural progenitor cell proliferation and survival	↑
Vitamins C and E levels	↓	regenerates the endogenous antioxidants, vitamins C and E → ↑ their antioxidant activity
Cholinergic activity	↓	↑
Mitochondrial function	↓	↑

**Table 2:** Histopathological scoring of Purkinje cell alterations in Wistar rats from the W-S, E-M, E-R, E-A, and E-S groups. Scoring criteria:—(normal: no visible alterations), + (mild: slight cell loss with occasional pyknosis and minor morphological changes), ++ (moderate: partial cell loss with more frequent pyknosis and moderate morphological changes), and +++ (severe: extensive cell loss with abundant nuclear pyknosis and pronounced morphological changes).

GROUP	PURKINJE CELL LOSS	NUCLEAR PYKNOSIS	MORPHOLOGICAL ALTERATIONS
Water–Saline (W-S)	-	-	-
Ethanol–Saline (E-S)	+++	+++	+++
Ethanol–Memantine (E-M)	+	+	+
Ethanol–Rivastigmine (E-R)	++	++	+
Ethanol–ALA (E-A)	++	+	+

## Data Availability

Data is contained within the article.

## Abbreviations

The following abbreviations are used in this manuscript:

ACh	Acetylcholine
AChE	Acetylcholinesterase
ALA	Alpha-lipoic acid
ARBD	Alcohol-related brain damage
BDNF	Brain-derived neurotrophic factor
CAT	Catalase
COX-2	Cyclooxygenase-2
DHLA	Dihydrolipoic acid
E-A	Ethanol–ALA group
EGs	Ethanol-drinking groups
E-M	Ethanol–Memantine group
E-R	Ethanol–Rivastigmine group
E-S	Ethanol–Saline group
GSH	Total glutathione
H&E	Hematoxylin and Eosin
IL	Initial latency
iNOS	Inducible nitric oxide synthase
LPO	Lipid peroxidation
NF-κB	Nuclear factor kappa B
NMDA	N-methyl-D-aspartate
NOX	Nicotinamide adenine dinucleotide phosphate oxidase
pCREB	cAMP Response Element-Binding Protein
PLA2	Phospholipase A2
ROS	Reactive oxygen species
SOD	Superoxide dismutase
STL	Step-through latency
TLR2	Toll-like receptor 2
TLR4	Toll-like receptor 4
TNFα	Tumor necrosis factor alpha
W-A	Water–ALA group
WGs	Water-drinking groups
W-M	Water–Memantine group
W-R	Water–Rivastigmine group
